# Epidemiological characteristics and pathogen profiles of non-*Escherichia coli* gram-negative urinary tract infections in pregnant women: insights from Makassar, Indonesia

**DOI:** 10.11604/pamj.2025.51.46.46208

**Published:** 2025-06-16

**Authors:** Kadri Rusman, Rizalinda Sjahril, Mochammad Hatta, Muhammad Nasrum Massi, Lisa Tenriesa Muslich, Fadhilah Syamsuri, Andi Meutiah Ilhamjaya

**Affiliations:** 1 Department of Clinical Microbiology, Faculty of Medicine, Hasanuddin University, Makassar, Indonesia,; 2 Department of Clinical Microbiology, Faculty of Medicine, Muhammadiyah University, Makassar, Indonesia,; 3 Clinical Microbiology Laboratory, Hasanuddin University Hospital, Makassar, Indonesia,; 4 Microbiology Laboratory, Wahidin Sudirohusodo Hospital, Makassar, Indonesia,; 5 Department of Clinical Microbiology, Faculty of Medicine, Alkhairaat University, Palu, Central Sulawesi, Indonesia

**Keywords:** Pregnancy, urinary tract infections, gram-negative bacteria, *Enterobacter cloacae*, microbial, epidemiology

## Abstract

**Introduction:**

urinary tract infections (UTIs) during pregnancy pose significant health risks for both mother and fetus. While Escherichia coli is the most common causative agent, non-Escherichia coli gram-negative bacteria also contribute to UTIs, with their specific prevalence and characteristics in pregnant women needing further elucidation, particularly in local contexts. This study aimed to identify gram-negative non-Escherichia coli bacteria causing UTIs in pregnant women and to determine their epidemiological characteristics in Makassar, Indonesia.

**Methods:**

a cross-sectional study was conducted at the Clinical Microbiology Laboratory of Hasanuddin University Hospital, Makassar, from July to August 2024. The study utilized 38 non-Escherichia coli bacterial isolates from urine cultures of pregnant women diagnosed with UTIs at various community health centers. After re-culturing on MacConkey Agar, bacterial identification was performed using the API 20e system. Epidemiological data were collected from medical records and questionnaires.

**Results:**

among 38 non-Escherichia coli isolates, Enterobacter cloacae was most prevalent (34.2%), followed by Klebsiella pneumoniae spp. Pneumoniae (23.7%). Most isolates (76.3%) were lactose fermenters. Epidemiologically, the 25-34 age group dominated (71.0%), with the highest UTI incidence in first pregnancies (47.4%) and the second trimester (44.7%). Significantly, 97.4% of pregnant women with UTI symptoms did not seek treatment.

**Conclusion:**

Enterobacter cloacae is the most common non-Escherichia coli gram-negative UTI bacterium in this Makassar cohort. Key epidemiological characteristics were elucidated, revealing a substantial proportion of women not seeking treatment for UTI symptoms. These findings underscore the importance of local surveillance and targeted health education interventions for pregnant women regarding UTI management.

## Introduction

Gram-negative bacteria are a significant public health concern globally due to their high resistance to various antibiotics [1]. These microorganisms, characterized by a thin peptidoglycan cell wall and an outer membrane containing lipopolysaccharide, appear pink or red after gram staining [2]. They have a substantial clinical impact in hospitals, potentially leading to intensive care unit admissions for high-risk patients and causing considerable morbidity and mortality [1]. *Enterobacteriaceae*, a diverse group of gram-negative bacteria, are widely distributed in nature and account for approximately 80% of gram-negative isolates that cause human diseases, including urinary tract infections (UTIs). Common *Enterobacteriaceae* affecting humans include *Escherichia coli, Proteus, Enterobacter, Klebsiella, Citrobacter, Yersinia, Shigella*, and *Salmonella*. Beyond *Enterobacteriaceae*, other clinically relevant gram-negative organisms include *Neisseria, Haemophilus spp, Helicobacter pylori*, and *Chlamydia trachomatis*. While non-fermenting gram-negative bacilli, such as *Pseudomonas aeruginosa, Acinetobacter baumannii, Burkholderia cepacia, Burkholderia pseudomallei, Stenotrophomonas, Alcaligenes*, and *Moraxella*, represent a smaller percentage of isolation compared to *Enterobacteriaceae* gram-negative bacilli, they are a relevant group causing severe opportunistic infections in hospitalized patients undergoing invasive treatment [3]. These bacteria are aerobic, non-spore-forming, and unable to ferment sugars, utilizing the oxidative pathway instead [4].

Urinary tract infection (UTI), defined as a disease caused by the invasion of microorganisms in the urinary tract from the renal cortex to the urethral meatus [5], is commonly observed in pregnant women [6,7]. Pregnancy increases the risk of UTI due to physiological changes, including ureteral dilation known as “hydronephrosis of pregnancy” that peaks around 22-26 weeks of gestation and continues until delivery. Increased levels of progesterone and estrogen during pregnancy also lead to decreased ureteral and bladder tone [8]. UTIs in pregnancy are categorized as symptomatic and asymptomatic bacteriuria [9]. Asymptomatic bacteriuria is defined as true bacteriuria (>100,000/ml) in the absence of specific symptoms of acute urinary tract infection, and has a prevalence of approximately 10% in pregnant women [9,10]. Untreated asymptomatic bacteriuria can progress to acute pyelonephritis, leading to higher maternal and neonatal morbidity. Therefore, a single urine screening for asymptomatic bacteriuria in the first trimester of pregnancy is crucial to mitigate the risk of acute pyelonephritis and its complications, while also limiting antibiotic exposure [11].

Accurate and rapid diagnosis of UTI is critical so that treatment can be given immediately to prevent perinatal complications such as bacteremia, premature birth, and low birth weight [12]. Quantitative urine culture, specifically using mid-stream specimen of urine (MSSU), is considered the gold standard for diagnosis, as it identifies the pathogen and tests its sensitivity, guiding clinical decisions [13]. A definitive diagnosis of UTI requires the isolation of a significant number of microorganisms, with ≥10^5^ CFU/ml obtained in cultured urine specimens being a significant criterion [14]. For bacterial identification from cultures, several biochemical tests can be performed. The Analytical Profile Index (API) identification method, specifically the API 20e microsystem, is a biochemical panel that has been used for the identification and differentiation of bacteria belonging to the *Enterobacteriaceae* family [15]. Introduced around 1970, the API 20e system uses conventional methods combined with new methods to produce better results and is accepted as a standard method whose results can be trusted by clinical microbiology laboratories. Its structure and chemical reactions on the API 20e strip have not changed since its introduction, allowing for comparison with historical research. Early studies, such as those by Darbandi *et al*. in 2011, demonstrated high accuracy rates (93% and 96.4%, respectively) for *Enterobacteriaceae* identification using the API 20e system [16].

Given the clinical significance of gram-negative non-*Escherichia coli* bacteria in pregnancy-related UTIs and the importance of accurate identification, the aim of this study was to identify gram-negative non-*Escherichia coli* bacteria and determine the epidemiological characteristics of pregnant women diagnosed with UTI from urine culture results with gram-negative non-*Escherichia coli* causative bacteria in Makassar, Indonesia.

## Methods

**Study design:** this study is a descriptive study with a cross-sectional study design.

**Study setting:** this study was conducted at the Clinical Microbiology Laboratory of Hasanuddin University Teaching Hospital, Makassar, during July-August 2024.

**Participants:** using the research subject 59 isolates from the urine culture results of pregnant women diagnosed with UTI at Tamalanrea Community Health Centers, Antara Community Health Centers, Kapasa Community Health Centers, Sudiang Raya Community Health Centers, and Tamalanrea Jaya Community Health Centers.

**Figure 1:**
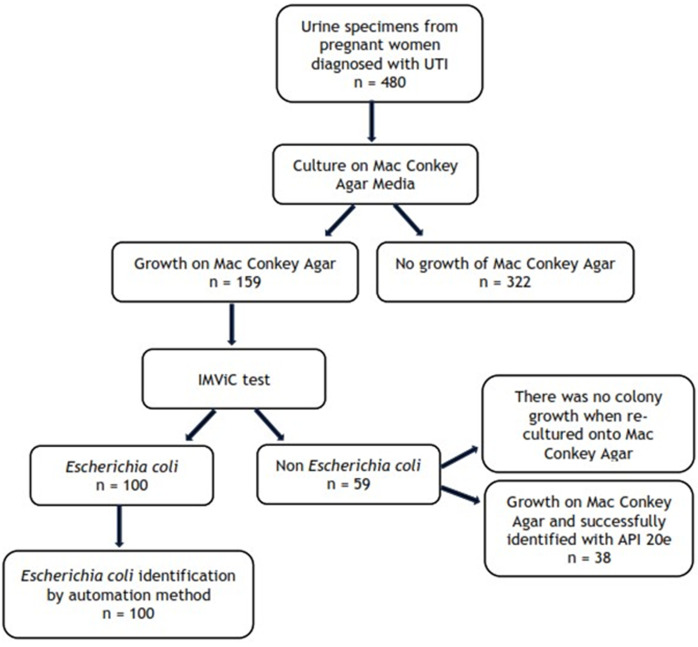
flowchart of isolated specimens used

**Research procedure:** this study used specimens in the form of isolates from previous studies that tested urine cultures of pregnant women diagnosed with UTI by comparing identification using Vitek 2 and Maldi-TOF MS which in previous studies only identified urine cultures of pregnant women with *Escherichia coli* results so we continued the study using isolates of urine culture results of non-*Escherichia coli* pregnant women. A total of 59 non-*Escherichia coli* isolates were refreshed and inoculated onto MacConkey Agar growth media, and only 38 isolates were found to have bacterial colony growth, followed by gram staining and identification by API 20e method (Figure 1).

**Variables:** the variables of this study were urine from pregnant women diagnosed with urinary tract infections and confirmed UTI in the Clinical Microbiology Laboratory of Hasanuddin University Teaching Hospital, Makassar from previous studies, and in the end only used 38 isolates that were successfully recultivated on MacConkey Agar media.

**Data source:** demographic data were collected through medical records and from questionnaires which included age, pregnancy history, pregnancy trimester, history of miscarriage, education and employment history, frequency of intercourse during the last three months, history of regular cleaning of the feminine area, history of vaginal discharge and medication history.

**Study size:** the sample size was determined using the Slovin formula [17].

**Inclusion and exclusion criteria:** this study used 38 isolates other than *Escherichia coli* from urine cultures of pregnant women who had been diagnosed with UTI by finding positive urine culture results of bacterial colonies >10^5^ cfu/ml at several community health centers in Makassar City in 2022-2023. Inclusion criteria of the study subjects were all collected isolates that grew on MacConkey media, and on indirect gram staining, a picture of gram-negative bacteria was found. Exclusion criteria of this study were all collected isolates that grew on MacConkey media and on indirect gram staining showed a picture of gram-negative bacteria, but were identified as *Escherichia coli* were not included as subjects of this study. Pregnant women with asymptomatic bacteriuria were also included in the exclusion criteria.

**Statistical methods:** data was processed using Microsoft Excel and analyzed with SPSS version 26 (Armonk, NY: IBM Corp.).

**Potential bias:** all bacterial identification tests using the API 20e system [15,16] were performed according to each manufacturer's protocol, and biochemical testing was previously performed using the IMVIC test and gram stain method, and we used the American Type Culture Collection (ATCC) isolate for *E. coli* as a quality control for bacterial identification.

**Ethical considerations:** the Ethics Team of Hasanuddin University has examined and approved this research. This research was conducted in accordance with all applicable requirements. Ethical approval was obtained from the Institutional Review Board of the Faculty of Medicine, Hasanuddin University (approval number: 448/UN4.6.4.5.31/PP36/2024, issued on June 14, 2024). All procedures followed the principles of the 1964 Declaration of Helsinki and its later amendments, Good Clinical Practice guidelines, and the International Conference on Harmonization standards.

## Results

**Participants:** a total of 38 non-*Escherichia coli* isolates were successfully recultured from urine specimens of pregnant women diagnosed with UTI, following initial identification processes as outlined in Figure 1.

**Descriptive data:** the majority of these isolates (76.3%) were lactose fermenters on MacConkey Agar, with the remaining 23.7% being non-lactose fermenters (Table 1).

**Table 1:** non-*Escherichia coli* isolates from the urine culture of pregnant women diagnosed with UTI on MacConkey Agar medium

No	MacConkey media culture	Isolate code	n
1	Lactose fermenter	76, 84, 91, 93, 95, 101, 105, 108, 110, 144, 148,164, 172, 174, 189, 194, 207, 215,216, 227, 232, 233, 257, 260, 267, 291, 296, 303	29
2	Non-lactose fermenter	68, 70, 120, 126, 168, 173, 249, 251, 424	9

UTI: urinary tract infection

**Outcome data:** among the identified gram-negative bacteria, *Enterobacter cloacae* was the most common cause of UTI, accounting for 34.2% of the isolates. *Klebsiella pneumoniae spp. Pneumoniae* was also frequently observed, making up 23.7% of the isolates (Table 2). For a complete breakdown of bacterial types, (Table 2).

**Table 2:** types of gram-negative bacteria from isolates identified using the API 20e identification method from urine cultures of pregnant women diagnosed with UTI in several health centers in Makassar City in 2022-2023

Types of gram-negative bacteria	n	%
*Enterobacter cloacae*	13	34.2
*Klebsiella pneumoniae spp pneumonia*	9	23.7
*Hafnia alvei*	3	7.9
*Serratia marcescens*	2	5.3
*Salmonella enterica ssp arizonae*	2	5.3
*Enterobacter aerogenes*	1	2.6
*Proteus mirabilis*	1	2.6
*Acinetobacter baumannii*	1	2.6
*Photobacterium damselae*	1	2.6
*Eikenella corrodens*	1	2.6
*Pantoea spp*	1	2.6
*Bordetella*	1	2.6
*Pseudomonas fluorescens*	1	2.6
*Enterobacter aerogenes*	1	2.6

UTI: urinary tract infection

**Main result:** epidemiological characteristics of the pregnant women with non-*Escherichia coli* UTIs showed several notable trends. The most prevalent age group was 25-34 years, comprising 71.0% of participants. Nearly half of the women (47.4%) were in their first pregnancy, and the highest incidence of UTI occurred in the second trimester (44.7%). Most participants (86.8%) had no history of miscarriage, and a majority (63.2%) had a college-level education. Furthermore, most pregnant women (73.7%) were not actively working during their pregnancy. Detailed epidemiological characteristics are presented in Table 3.

**Table 3:** epidemiologic characteristics of pregnant women diagnosed with UTI from culture results identified as non-*Escherichia coli*

Characteristics of pregnant women diagnosed with UTI (urinary tract infection)	n	%
**Age (years)**		
18-24	6	15.8
25-34	27	71.0
35-44	5	13.2
**Pregnancy history**		
First pregnancy	18	47.4
Second pregnancy	11	28.9
Greater than third pregnancy	9	23.7
**Trimester of pregnancy**		
First trimester	11	29.0
Second trimester	17	44.7
Greater than third pregnancy trimester	10	26.3
**History of miscarriage**		
Yes	5	13.2
No	33	86.8
**Educational history**		
Elementary school	1	2.6
Junior high school	2	5.3
Senior high school	11	28.9
College	24	63.2
**Work history**		
Work	10	26.3
Not working	28	73.7
**Frequency of intercourse between husband and wife in the last 3 months**		
Less than 3 x/week	24	63.2
> < 3 x/week	8	21.0
Never	6	15.8
**Cleaning the feminine area regularly**		
Yes	27	71.1
No	11	28.9
**Treatment history with presenting complaints**		
Get treatment	1	2.6
No treatment	37	97.4
**History of current vaginal discharge**		
Yes	6	15.8
No	32	84.2

**Other analyses:** in terms of lifestyle and health behaviors, 63.2% of participants reported a frequency of sexual intercourse less than three times per week in the last three months. A large majority (71.1%) reported regularly cleaning the feminine area. Notably, a significant proportion (97.4%) of pregnant women with UTI symptoms did not seek treatment for their complaints, and most (84.2%) did not present with a history of current vaginal discharge (Table 3).

## Discussion

This study used isolates isolated from urine cultures of pregnant women diagnosed with UTI from previous studies. In previous studies, using urine cultures believed to be *Escherichia coli* with several tests, namely from looking at the morphology of colonies that grow on MacConkey media, so that the characteristics of *Escherichia coli* colonies on MacConkey Agar media are pink, non-mucoid colonies with lactose fermentation properties and IMViC tests (Indole positive, Methyl Red positive, Voges-Proskauer and Citrate negative) and identification tests using VITEK 2 and MALDI-TOF MS with the results identified as *Escherichia coli*. So that 59 non-*Escherichia coli* isolates were found to grow on MacConkey media that with colony characteristics and IMViC test results, were declared as non-*Escherichia coli* isolates. A total of 38 non-*Escherichia coli* isolates were used as subjects in this study, and carried out bacterial identification with the API 20e system, and analyzed the epidemiological characteristics of research subjects diagnosed with UTI with culture results showing gram-negative non-*Escherichia coli* bacteria. We did not include *Escherichia coli* because the culture results from the urine of pregnant women diagnosed with UTI, with *Escherichia coli* identification results, have been carried out by previous researchers.

This study also assessed the epidemiological characteristics of pregnant women diagnosed with UTI, with urine culture results showing gram-negative non-*Escherichia coli* bacteria. Our research obtained different results from research conducted by Fakhrizal *et al*. obtaining different results from the characteristics of gestational age, the highest incidence of UTI in the third trimester of pregnancy was 55.6%, and had the same results as our research in terms of work history characteristics where the highest incidence of UTI in pregnant women who did not have a job was 73.7% [18]. The same thing from our research with research conducted by Johnson *et al*. found that the incidence of UTI in pregnant women was highest at the age of 25-34 years as much as 21.2% and from the characteristics of education history as much as 7.9% had education up to college, the same results were also obtained in the characteristics of pregnancy history, the incidence of UTI in pregnant women was highest in the first pregnancy at 11.7% [19]. From this study, it can be said that higher education does not guarantee that a pregnant woman can avoid urinary tract infections, and a person with a first pregnancy is more susceptible to UTIs.

The results of the identification of 38 non-*Escherichia coli* isolates using the API 20e system obtained *Enterobacter cloacae* (34.2%) as the most bacteria that cause UTI, in contrast to research conducted by Rosana *et al*. getting the most results of bacteria that cause UTI besides *Escherichia coli* is *Klebsiella pneumoniae* as much as 20% [20], and research by Nufaliana *et al*. found *Pseudomonas aeruginosa* as much as 38.46% as the most gram-negative bacteria after *Escherichia coli* [21].

The dominance of *Enterobacter cloacae* as a non-*E. coli* gram-negative pathogen in pregnant women with UTIs carries significant clinical implications. As a member of the ESKAPE group of bacteria, *Enterobacter cloacae* is notorious for its ability to develop resistance to a wide array of antibiotics, posing a substantial challenge to effective treatment [22]. This intrinsic and acquired resistance, including the production of AmpC β-lactamases and extended-spectrum beta-lactamases (ESBLs), can lead to treatment failures with commonly prescribed antibiotics like ampicillin, first-generation cephalosporins, cefoxitin, and amoxicillin-clavulanate [23,24]. Such resistance necessitates careful antimicrobial susceptibility testing to guide appropriate therapeutic choices and prevent the escalation of infection.

The clinical significance is further amplified in pregnant women due to the heightened risks associated with UTIs during gestation. Untreated or inadequately treated UTIs in pregnant women can lead to severe maternal and neonatal morbidity and mortality, including progression to acute pyelonephritis, which is a major cause of septic shock in this vulnerable population [25,26]. Furthermore, *Enterobacter cloacae* infections, particularly those with resistant strains, are linked to adverse pregnancy outcomes such as preterm delivery and low birth weight. The potential for perinatal transmission of resistant Enterobacterales from colonized mothers to neonates is also a serious concern, as these infections can result in increased neonatal mortality, morbidity, and healthcare costs [27]. Therefore, the prevalence of *Enterobacter cloacae* underscores the critical need for robust surveillance of local antibiotic resistance patterns. Timely and accurate identification of this pathogen, coupled with precise antimicrobial susceptibility testing, is paramount for guiding empiric and definitive antibiotic therapy. This approach is essential to minimize the risk of complications for both the mother and the neonate, mitigate the spread of multidrug-resistant strains, and improve overall pregnancy outcomes.

**Limitations:** the limitation of our study was the presence of 21 out of 59 isolates of gram-negative non-*Escherichia coli* bacteria that could not grow after re-culturing from Brain Heart Infusion Broth to MacConkey Agar, so the data on the cause of UTI in pregnant women with gram-negative non-*Escherichia coli* bacterial infection is limited. Our bacterial identification uses biochemical methods with the API 20e system instead of automated methods with machines to help health care centers such as small hospitals that cannot afford to hold clinical microbiology laboratories with modern identification tools using automated identification machines to be able to start holding clinical microbiology laboratory services in hospitals using bacterial identification with simple and more economical methods and with reliable results using the API 20e system for gram-negative bacteria of the *Enterobacteriaceae* group and there are still some examinations from API for other gram-negative and also for gram-positive bacteria.

## Conclusion

This study concluded that among the 38 non-*Escherichia coli* isolates from urine cultures of pregnant women diagnosed with urinary tract infections (UTIs), *Enterobacter cloacae* was the most prevalent gram-negative bacterium causing UTIs. The highest incidence of UTIs was observed in first pregnancies and during the second trimester. Epidemiological analysis also revealed a higher occurrence in pregnant women aged 25-34 years, those with a college-level education, and individuals who were not actively employed during pregnancy. A significant finding was that 97.4% of pregnant women experiencing UTI symptoms did not seek treatment. These results highlight specific epidemiological patterns of non-*Escherichia coli* UTIs in the studied pregnant population and indicate a substantial gap in healthcare-seeking behavior.

### What is known about this topic

- Gram-negative bacteria are common microorganisms, often antibiotic-resistant, posing significant public health concerns due to associated morbidity and mortality;
- Urinary tract infections are highly prevalent in pregnant women, largely due to physiological changes during gestation that increase susceptibility;
- Asymptomatic bacteriuria is common during pregnancy and can progress to more severe pyelonephritis if left untreated. Quantitative urine culture is the diagnostic gold standard for UTIs. Biochemical methods like the API 20e system are established and reliable for bacterial identification.

### What this study adds

- Identified Enterobacter cloacae as the most common non-E. coli gram-negative UTI pathogen (34.2%) in pregnant women;
- Provided specific epidemiological characteristics of non-E. coli UTIs in this population, including age, pregnancy history, and education;
- Revealed a significant gap in healthcare access, with 97.4% of pregnant women with UTI symptoms not seeking treatment. Contributes local data for Makassar, aiding in understanding regional pathogen prevalence and patient profiles.
